# Clinical Adjustment of Zirconia-Reinforced Lithium Silicate and Lithium Disilicate Restorations Should Be Performed Before Crystallization

**DOI:** 10.3390/ma18091944

**Published:** 2025-04-24

**Authors:** Kusai Baroudi, Nathália Ribeiro de Almeida, Laura Salerno de Abreu, Vinícius Felipe Wandscher, Nathalia de Carvalho Ramos, Vivek Padmanabhan, Caroline Andrade Bucholz, Marina Amaral

**Affiliations:** 1 Department of Clinical Sciences, College of Dentistry, Ajman University, Ajman P.O. Box 346, United Arab Emirates; d_kusai@yahoo.co.uk; 2 Department of Dentistry, Universidade de Taubate, Taubate 12020-270, Brazil; nathaliaribalm@gmail.com (N.R.d.A.); laah.salerno@gmail.com (L.S.d.A.); caroline.abmatos@unitau.br (C.A.B.); 3 Prosthodontics Unit, Faculty of Dentistry, CNEC Faculty of Santo Ângelo, Santo Ângelo 98801-015, Brazil; viniwan@hotmail.com; 4 Department of Dental Materials and Prosthodontics, Institute of Science and Technology, São Paulo State University (Unesp), São José dos Campos 12245-000, Brazil; nathalia.carvalhoramos@gmail.com; 5 Pediatric and Preventive Dentistry, RAK College of Dental Sciences, RAK Medical and Health Sciences University, Ras Al-Khaimah P.O. Box 2990, United Arab Emirates; vivek.padmanabhan@rakmhsu.ac.ae

**Keywords:** lithium disilicate, zirconia-reinforced lithium silicate, abrasion, surface roughness, biaxial flexural strength, dental ceramics

## Abstract

The objective of this study was to evaluate the biaxial flexural strength of zirconia-reinforced lithium silicate (ZLS, Suprinity, Vita Zahnfabrik, Bad Säckingen, Germany) and lithium disilicate (LDS, IPS e.max CAD, Ivoclar Vivadent, Schaan, Liechtenstein) discs after abrasion with a diamond tip, before or after crystallization of the ceramic. Discs of 1.2 × 15 mm dimensions were fabricated. The samples were separated into two groups: AC—abrasion with a diamond tip before material crystallization, and CA—material crystallization and subsequent abrasion with a diamond tip (*n* = 15). The initial roughness was measured before abrasion/crystallization, and final measurement was performed after abrasion/crystallization/polishing. The abraded surface was placed downward during the biaxial flexural strength test, and the data were recorded. The final roughness was significantly higher compared to the initial roughness in all groups. The ZLS-AC and LDS-AC groups (both materials with abrasion prior to material crystallization) showed higher biaxial flexural strength values than groups that underwent abrasion before crystallization. This study concluded that the clinical adjustment performed by abrasion with the diamond tip of glass ceramics lithium disilicate and zirconia-reinforced lithium silicate carried out prior to crystallization favored the resistance of the ceramics.

## 1. Introduction

Lithium disilicate (LDS) and zirconia-reinforced lithium silicate (ZLS) ceramics have become widely used in restorative dentistry due to their excellent combination of mechanical strength, esthetics, and clinical versatility. Monolithic LDS crowns have been successfully applied in dentistry since 1998 (IPS Empress 2, Ivoclar Vivadent), and have demonstrated minimal clinical complications, achieving 100% survival rate after five years [1]. Furthermore, the survival rate of LDS restorations has been reported at 96.8% after an average observation period of 7.8 ± 3.3 years, with an annual failure rate of 0.2%. Reasons for failure included secondary caries, restoration fractures, and endodontic complications [2].

Comparative studies indicate that ZLS crowns exhibit fracture resistance values comparable to those of LDS crowns, where both materials meet clinically acceptable thresholds for posterior restorations [3]. ZLS studies are available since 2015 and can be considered promising hybrid ceramic materials for CAD-CAM technologies [4]. The ZLS is composed by a glassy matrix, with crystalline components of lithium metasilicate and orthophosphates, in addition to tetragonal zirconia filler, in an attempt to improve strength values [4]. More basic and clinical studies are necessary to define the mechanical and biological behavior of ZLS.

Nevertheless, clinical adjustments of ceramic restorations are frequently required before cementation, regardless of the impression and fabrication methods [5]. Adjustments using diamond rotary instruments result in significant surface damage and increased surface roughness [6]. These procedures have been linked to reduced fatigue failure loads compared to unadjusted restorations [7]. Clinically, such adjustments are usually performed after crystallization, in the same section of cementation.

Material composition may play a critical role in the mechanical properties of these ceramics. Pre-crystallized lithium silicate contains approximately 40% primary lithium metasilicate (Li_2_SiO_3_) crystals and secondary lithium orthophosphate (Li_3_PO_4_) crystals, with grain sizes ranging from 0.2 to 1 mm embedded in a glass matrix [8]. The material’s silica content varies between 57% and 80% by weight, with lithia (11–19 wt%) and smaller amounts of potassium, phosphorus pentoxide, zirconia, and zinc oxide [9]. This composition yields a biaxial strength of approximately 130 ± 30 MPa and fracture toughness of 1.29 ± 0.06 MPa.m1/2 [10]. Pre-crystallized ZLS silicate features dual crystal phases—sub-micron lithium metasilicates and nanometric lithium orthophosphates—designed for efficient CAD/CAM processing [10]. The material demonstrates a biaxial strength of 180 MPa, a fracture toughness of 0.91 ± 0.05 MPa.m1/2, and a Poisson’s ratio of 0.21 [11]. Both ceramic types are milled in pre-crystallized form. After milling, the crystallization changes the structure of the materials, providing improved mechanical and optical properties. LDS becomes a crystalline material with about 70% of crystals of orthorhombic form of lithium disilicate (Li_2_Si_2_O_5_), while ZLS presents a multicomponent structure, going from nucleation into the melty glass phase, where zirconia particles cannot be identified, making it part of the crystalline network [12].

Surface treatments significantly influence the properties of glass ceramics. Abrasive procedures, such as grinding with diamond burs, decrease the flexural strength of LDS ceramics [13]. A previous study showed that polishing the previously abraded occlusal surface of LDS restorations enhances the survival rate of restorations [14]. Polishing and glazing are effective methods to reduce surface roughness, with glazed ceramics demonstrating higher flexural strength and smoother surfaces compared to other finishing methods [15]. Nevertheless, the flexural strength of CAD-CAM glass ceramics can be compromised by milling, etching, and fitting adjustments. The ceramic microstructure, etching protocol, and hydrofluoric acid concentration determine the impact on mechanical properties and surface roughness [16].

Some studies indicate that polishing may promote smoother and more uniform surfaces than glazing, with comparable flexural strength, and some polishing systems associated with alterations on surface roughness may promote a decrease in ceramic flexural strength [17]. Furthermore, glaze firing after surface defect incorporation has been shown to repair strength in LDS ceramics, although this effect is not consistently observed in ZLS materials [18].

Considering the mechanical and surface property challenges associated with LDS and ZLS ceramics, the timing of surface adjustments is a critical factor. Defects introduced after crystallization could negatively impact strength, whereas those introduced before crystallization might have less detrimental effects [18]. Thermal treatment after surface alterations has been demonstrated to heal defects which could lead to a decrease in the strength of the material [12]. Additionally, morphological changes due to polishing affect fatigue performance, requiring careful handling during marginal adjustments [4]. Biaxial flexural strength has been used as the standard test for evaluating the strength of brittle materials [19], since they tend to be weaker in tension than in compression.

Thus, the present study aims to evaluate the biaxial flexural strength of lithium disilicate and zirconia-reinforced lithium silicate ceramic discs after simulated adjustments using diamond burs, performed either before or after ceramic crystallization. The null hypothesis tested is that the moment of ceramic crystallization does not affect the strength of the tested materials.

## 2. Materials and Methods

Prefabricated blocks of LDS (IPS e.max CAD, Ivoclar Vivadent, Schaan, Liechtenstein) and ZLS (Suprinity, Vita Zahnfabrick, Bad Säckingen, Germany) were used. The blocks were manually rounded into cylinders with a diameter of 15 mm using a bench-top polisher (Aropol E, Arotec, Lower Hutt, New Zealand) with 400-grit silicon carbide paper. The cylinders were then mounted on a precision cutting machine (ISOMET, Buehler, Lake Bluff, IL, USA) and sectioned using a diamond blade under water cooling to obtain discs with a thickness of 1.2 mm (±0.2 mm). The discs were finished on both sides using a bench-top polisher (Aropol E, Arotec) with 600-grit silicon carbide paper.

The samples were initially subjected to surface roughness (Ra) measurements: three surface readings were performed in parallel directions using a contact profilometer (SurfTest SJ-310, Mitutoyo, Kanagawa, Japan) with a 0.25 mm cutoff and a speed of 0.5 mm/s. The average roughness of each disc (Ra—μm) was recorded.

The samples were then divided into 2 groups by material (*n* = 15): Group 1—diamond bur abrasion **before** ceramic crystallization (ZLS-AC, LDS-AC); Group 2—diamond bur abrasion **after** ceramic crystallization (ZLS-CA, LDS-CA).

For the AC samples (abrasion → crystallization), one surface of the disc was marked with a permanent marker, and diamond bur abrasion was performed using a cylindrical extra-fine-grit diamond bur (3099 FF, KG Sorensen, Miami, FL, USA) mounted on a high-speed handpiece under manual pressure until the marker was completely removed from the surface. Any standardization in this procedure was discarded to represent a more realistic clinical adjustment. Abrasion was followed by surface polishing with silicone diamond discs (sequence of granulation DF to DFF, indicated for finishing and smoothening by manufacturer, 8090 D, Viking, KG Sorensen) in side-to-side movements, for one minute. The samples were then subjected to crystallization in a furnace (Programat EP 5000, Ivoclar Vivadent, Schaan, Liechtenstein). After crystallization, final surface roughness measurements were performed, as described before.

For the CA samples (crystallization → abrasion), crystallization was performed first (Programat EP 5000, Ivoclar Vivadent). Then, one surface of the disc was marked with a permanent marker, and diamond bur abrasion was performed using a cylindrical extra-fine-grit diamond bur (3099 FF, KG Sorensen) mounted on a high-speed handpiece under manual pressure until the marker was completely removed from the surface. Abrasion was followed by surface polishing with rubber points (Vicking, KG Sorensen) for one minute. Final surface roughness measurements were then performed as described before.

To evaluate material strength, the biaxial flexural strength test was applied. The samples were positioned on a universal testing machine (MBio, 5000, BIOPDI, São Carlos, Brazil) with a base containing three metallic spheres, each 1.6 mm in diameter, arranged in a 10 mm diameter circle, 120° apart from each other. The surface subjected to diamond bur abrasion was placed facing downward during the flexural strength test. A cylindrical metal tip (1.6 mm in diameter) with a flat end applied an increasing load (0.5 mm/min) until sample fracture. The maximum load (N) was recorded for subsequent calculation of biaxial flexural strength (Equation (1)):

(1) $$S=-0.2387\;\frac{P(X-Y)}{d^{2}}$$

where *S* = maximum stress, in MPa; *P* = total load required to cause fracture, in Newtons; *d* = sample thickness at the fracture origin, in millimeters.

The samples were evaluated for the number of fragments and fracture origin.

The Weibull parameter module ( m) and the characteristic strength ( $\sigma_{0}$ ) were determined in a $ln\sigma_{0\;}-ln[1/(1-F\left(\sigma_{0}\right)]$ diagram (according to ENV 843-5 [20]):

$$\ln\ln\frac{1}{1-F_{\left(\sigma_{0}\right)}}=m\ln\sigma_{c}-m\ln\sigma_{0}$$

### Statistical Analysis

All groups were individually assessed for normality using the Kolmogorov–Smirnov test. For roughness analysis, each group was compared for initial roughness and final roughness using the paired *t*-test. The increase in roughness between initial roughness and final roughness for each sample was calculated, and increase in roughness values was compared between materials using an independent sample *t*-test. All statistical tests were performed at Minitab (v.17, Minitab Inc., State College, PA, USA). Representative images of group surfaces were performed by scanning electron microscopy.

Power analysis was calculated as 1, considering 4 levels, α=0.05 , sample size *n* = 15, mean standard deviation of results (34.22), and maximum difference between groups = 129 MPa. For biaxial flexural strength analysis, an independent sample *t*-test was initially performed to compare abrasion/crystallization moment for each material. Two-way ANOVA (material and abrasion/crystallization timing) was then performed, followed by Tukey’s post hoc test for pairwise comparisons among all groups. Fracture analysis was performed after the test, considering the number of fragments per sample and failure origin. Representative images were performed by scanning electron microscopy.

## 3. Results

**Figure 1:**
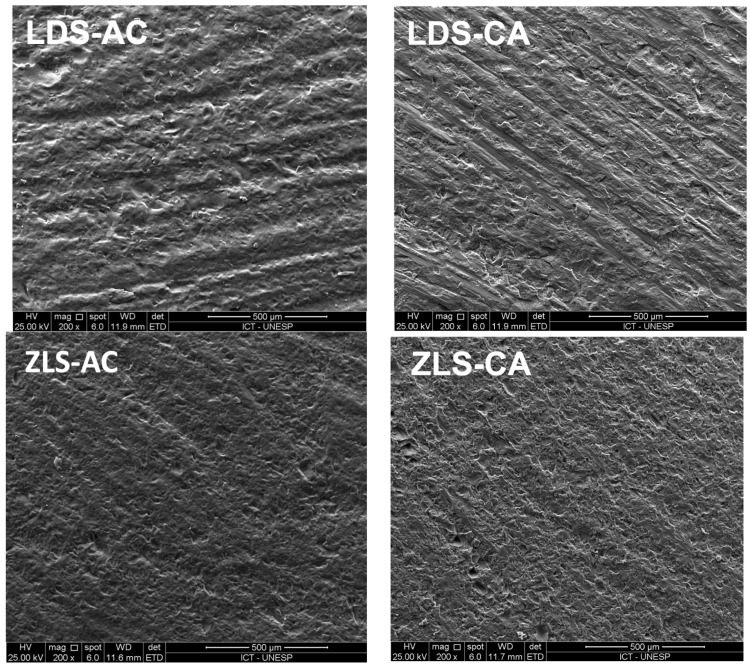
Scanning electron microscopy images of each group. LDS-AC (lithium disilicate abraded and then crystallized); LDS-CA (lithium disilicate crystallized and then abraded); ZLS-AC (zirconia-reinforced lithium silicate abraded and then crystallized); ZLS-CA (zirconia-reinforced lithium silicate crystallized and then abraded)—magnification 200 × .

All groups showed normal distribution when subjected to the Kolmogorov–Smirnov test. A significant increase in roughness was observed between the initial and final measurements for all tested conditions and both materials (Table 1). There was no significant difference in the increase in roughness (final Ra—initial Ra) regarding material, considering the roughness increase in each abrasion/crystallization scenario (Table 2). Figure 1 shows representative scanning electron microscopy images of each group.

For biaxial flexural strength, the *t*-test indicated a significant difference within each material depending on the sequence of crystallization and abrasion. For both materials, the strength was significantly lower when abrasion was performed after crystallization (Table 3).

Table 4 presents the results of the two-way ANOVA test (material × moment of crystallization), showing a significant influence only for the moment of abrasion and crystallization. In Table 5, the group comparison analysis clearly indicates that abrasion after crystallization resulted in lower biaxial flexural strength values for both materials.

Weibull analysis revealed (Table 6) that reliability values ( m ) were similar between all groups, indicated by the overlap between confidence intervals (CIs), and that there was a significant decrease in characteristic strength ( $\sigma_{0})$ when materials were first crystallized and then adjusted.

**Figure 2:**
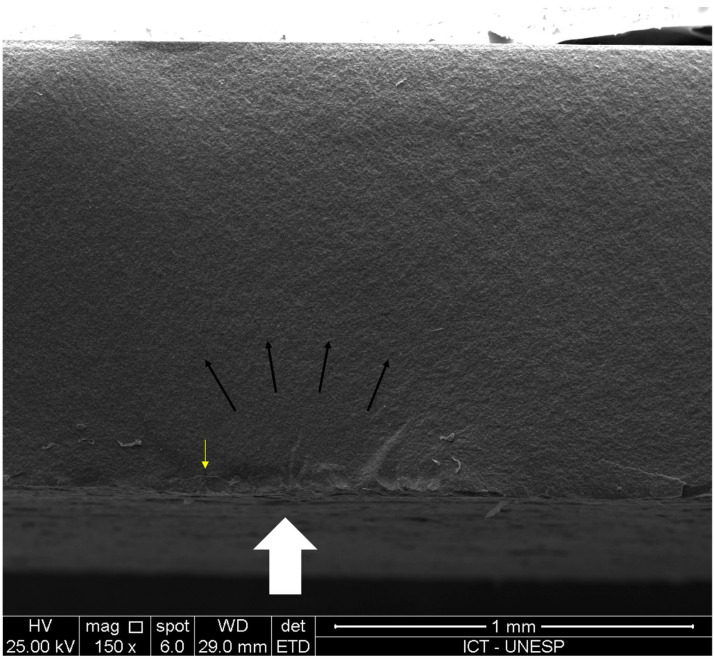
Representative scanning electron microscopy of LDS-CA (lithium disilicate crystallized and then abraded) sample at fracture origin (white arrow), with subsurface cracks caused by abrasion (yellow arrow); black arrows indicate the direction of fracture propagation (500 × magnification).

The fracture analysis showed more fragments associated with the highest flexural strength values (minimum of 2 fragments from 50 MPa to 150 MPa, and maximum of 6 fragments at 280 MPa). All factures had their origin at the abraded surface (which was under tensile stress during the test). Figure 2 shows a fracture origin showing a rough surface and subsurface cracks caused by abrasion in LDS abraded after crystallization (DCA).

## 4. Discussion

Based on the results of this study, it is evident that regardless of the moment of diamond bur abrasion and crystallization, even when followed by polishing, the roughness of both ZLS and LDS ceramics is affected, showing higher values after abrasion (Table 1). For biaxial flexural strength, abrasion after crystallization resulted in the lowest flexural strength values for both materials (Table 5). Thus, the null hypothesis was rejected.

The values obtained for flexural strength from both materials were lower than the predicted values obtained for the materials without surface alteration. Biaxial flexural strength values for LDS reported in the literature varied between 343.57 MPa and 378.88 MPa [21,22,23], which are lower than the reported values in the present study even when abrasion was performed before crystallization: 244.3 MPa (Table 3). Values of biaxial flexural strength for ZLS reported in the literature are around 289.1 MPa [24], reaching values as high as 507.53 MPA [23], which were also higher than the data obtained in the present study, ZLS-AC = 232.1 MPa and ZLS-CA = 115.0 MPa (Table 3). This demonstrates that abrasion damage was irreversible, even when followed by polishing and crystallization.

Clinically, performing diamond bur adjustments before crystallization extends one clinical session, as the restoration must be returned to the laboratory for crystallization. In contrast, when adjustments are made after crystallization, the dentist can polish the adjusted area and proceed with cementation. However, this approach compromises the material’s strength. The chairside workflow has an advantage in such a scenario, since the adjustment is fast and simple and can be performed after restoration milling. Then, the crystallization cycle is performed and restoration is installed clinically.

It is important to note that despite the polishing performed in this study, final surface roughness values were higher (Table 1), indicating unrepaired surface damage in both ceramics. Surface damage in glass ceramics, such as those tested in this study, creates stress concentration sites where the critical strength value is reduced, and these defects are likely fracture origins, leading to fracture of the prosthetic piece even under loads below the critical strength of the material [18]. These surface defects left by abrasion, which can be aggravated in the oral environment by contact with the antagonist (fatigue wear), play an important role in the longevity of restorative treatments with ceramics [4]. The application of a glaze layer after adjustment may be an alternative to heal the surface defects and prevent the decrease in LDS strength [15].

LDS has been considered the gold standard for adhesive restorations due to its strength, esthetics, and ease of adhesive cementation, with high clinical survival rates [1]. ZLS is also recommended to be adhesively bonded to the substrate, and is less prone to decrease in strength due to acid etching when compared to LD [25,26]. Cementation protocol of both ceramics include 5% hydrofluoric acid etching during 20 s [26]. ZLS incorporates zirconia reinforcement, added by manufacturers to improve the material’s fracture toughness. In the present study, regarding alteration surface by abrasion, both materials exhibited similar strength values (Table 5) and demonstrated comparable behavior under the tested crystallization/abrasion protocols.

The mechanism underlying the higher strength values observed in the groups where abrasion was performed before crystallization likely involves partial sealing of surface defects caused by the melting of the material’s glassy phase during crystallization. Previous studies have shown a reduction in the size of cracks and surface defects after LDS crystallization [18] or even difficulty in detecting surface defects introduced before crystallization under scanning electron microscopy [11]. Similar phenomena may be the reason for the highest strength when abrasion was performed before crystallization in both glass ceramics tested in this study. Figure 1 shows the fracture origin of a sample which had its surface abraded after crystallization, with subsurface cracks, contributing to flexural strength decrease. Damages to the surface are also shown in Figure 1, where samples abraded after crystallization (left column) have apparently sharper damages than samples abraded before crystallization (right column).

In this study, glaze application was not simulated, but previous research indicates that it is even more effective in fully restoring the material’s strength after abrasion [18] for specific brands of LDS. The effect of glazing would be complete sealing of surface defects. Although the firing process alone has advantages in restoring material strength, glaze application or reapplication is more efficient [18].

In terms of damage repair, ZLS might theoretically offer an advantage due to the toughening mechanism provided by zirconia. However, this advantage was not observed in increased strength or resistance to slow crack growth due to the presence of zirconia [8]. In the present study, the presence of zirconia also did not yield better results, as ZLS showed similar outcomes to LDS regardless of whether the abrasion was performed before or after crystallization (Table 5).

Regarding Weibull analysis (Table 6), the results were similar to what was reported before. Reliability was higher for LDS than for ZLS, but the results were considered similar by the overlap of confidence intervals. This analysis is applied to the analysis of strength of materials, mainly related to ceramics. The characteristic strength was considered to be the strength at a failure probability of approximately 63.2%. This represents a practical estimation of the mean strength of the material. The Weibull module was used as a measure of the distribution of strength data, which expresses the reliability/structural homogeneity of the material. A high (m) value indicates low data dispersion, which means that the material presents a more consistent behavior in terms of strength. A low (m) value indicates high variability regarding internal defects of ceramics and, consequently, more uncertainty in strength [27].

According to the results of this study, performing clinical trials and adjustments of the prosthetic piece before the final crystallization would be beneficial for the strength of the tested materials. However, final crystallization of the material increases the strength, hardness, elastic modulus, fracture toughness, and flexural strength of ZLS [4]. Therefore, utmost care should be taken if the clinician opts to perform the trial and adjustments before crystallization.

This is a preliminary pilot study, and future research based on these findings should be conducted. The limitations of this study include the evaluation of ceramics of geometric shapes, which distribute loads differently from clinical crown shapes. Regarding roughness evaluation, the present study only considered Ra parameter for analysis, since it is a basic variable for evaluation of roughness, but it is recommended to evaluate at least one more parameter (Sa, Sz, Rt) for the description of roughness in ceramic samples; if possible, a 3D profilometer analysis would bring more insights regarding surface profile. This study also did not assess ceramics subjected to aging processes or repeated loads in a humid environment, as occurs in the oral cavity. Glaze re-application after adjustments should be considered, especially in cases where the adjustment is performed after crystallization.

## 5. Conclusions

It is possible to conclude that the clinical adjustment of ceramics based on lithium disilicate (LDS) and zirconia-reinforced lithium silicate (ZLS) should be performed prior to crystallization for better mechanical strength.

## Figures and Tables

**Table 1:** Paired *t*-test result for roughness analysis in each group.

Group	Initial Roughness	Final Roughness	*p*-Value
ZLS-AC	0.763 (0.078)	2.780 (0.748)	*p* = 0.000
ZLS-CA	0.9910 (0.2718)	2.6849 (0.3678)	*p* = 0.000
LDS-AC	0.785 (0.125)	3.540 (0.911)	*p* = 0.000
LDS-CA	0.8282 (0.170)	3.157 (1.207)	*p* = 0.000

ZLS-AC: zirconia-reinforced lithium silicate abraded and then crystallized; ZLS-CA: zirconia-reinforced lithium silicate crystallized and then abraded; LDS-AC: lithium disilicate abraded and then crystallized; LDS-CA: lithium disilicate crystallized and then abraded.

**Table 2:** Comparison of increase in roughness between materials.

Increase in Roughness			*p*-Value
Final Ra roughness—initial Ra roughness	ZLS-AC = 2.006 (0.772)	ZLS-CA = 1.651 (0.673)	*p* = 0.191
	LDS-AC = 2.800 (0.980)	LDS-CA = 2.40 (1.35)	*p* = 0.366

ZLS-AC: zirconia-reinforced lithium silicate abraded and then crystallized; ZLS-CA: zirconia-reinforced lithium silicate crystallized and then abraded; LDS-AC: lithium disilicate abraded and then crystallized; LDS-CA: lithium disilicate crystallized and then abraded.

**Table 3:** Mean and standard deviation for biaxial flexural strength comparing the moment of crystallization for each material (independent *t*-test).

Material	Moment of Crystallization *		*p*-Value
ZLS	AC = 232.1 (56.2)	CA = 115.0 (27.1)	*p* = 0.000
LDS	AC = 244.3 (35.7)	CA = 135.2 (17.9)	*p* = 0.000

*AC: first abrasion, followed by crystallization; CA: first crystallization, followed by abrasion.

**Table 4:** Two-way analysis of variance considering the material and the moment of crystallization as factors.

Source	DF	Adj SS	ADJ MS	F-Vale	*p*-Value
Material	1	3939	3939	2.87	0.096
Moment of crystallization	1	191,810	191,810	139.91	0.000
Material × Moment of crystallization	1	236	236	0.17	0.680
Error	56	76,772	1371		
Total	59	272,758			

**Table 5:** Mean comparison by Tukey’s test.

Group	N	Mean	Grouping
LDS-AC	15	244.32	A
ZLS-AC	15	232.08	A
LDS-CA	15	135.21	B
ZLS-CA	15	115.03	B

ZLS-AC: zirconia-reinforced lithium silicate abraded and then crystallized; ZLS-CA: zirconia-reinforced lithium silicate crystallized and then abraded; LDS-AC: lithium disilicate abraded and then crystallized; LDS-CA: lithium disilicate crystallized and then abraded.

**Table 6:** Weibull analysis results for reliability ( m ) and characteristic strength ( $\sigma_{0})$ .

Group	m	m CI (95%)	$\sigma_{0}$	$\sigma_{0}$ CI (95%)
ZLS-AC	3.741	(2.160–5.225)	259.15	(217.63–308.39)
ZLS-CA	4.254	(2.456–5.941)	126.79	(108.74–147.75)
LDS-AC	7.270	(4.197–10.154)	259.36	(237.08–283.66)
LDS-CA	8.425	(4.864–11.767)	143.14	(132.46–154.64)

ZLS-AC: zirconia-reinforced lithium silicate abraded and then crystallized; ZLS-CA: zirconia-reinforced lithium silicate crystallized and then abraded; LDS-AC: lithium disilicate abraded and then crystallized; LDS-CA: lithium disilicate crystallized and then abraded.

## Data Availability

The data presented in this study are available on request from the corresponding author.
